# Cancer Prevention and Therapy With Nutritional Science: Addressing the Gap in Medical Education and Practice

**DOI:** 10.1002/mco2.70417

**Published:** 2025-10-15

**Authors:** Taylor E. Collignon, Anupam Bishayee

**Affiliations:** ^1^ Department of Pharmacology College of Osteopathic Medicine Lake Erie College of Osteopathic Medicine Bradenton Florida USA

**Keywords:** cancer, carcinogens, diet, nutrition, osteopathic medicine, prevention

## Abstract

Cancer is a leading cause of morbidity and mortality worldwide. There exists a correlation between certain cancers and dietary factors. Several known carcinogens are present in the standard American diet, also known as the Western Diet. Additionally, food preparation methods can initiate carcinogenesis. Various dietary components, particularly plant‐based foods, contain bioactive phytochemicals, have demonstrated potential anticancer effects through various molecular mechanisms. Consuming a wide variety of these so‐called “cancer‐fighting” foods may lead to synergism in preventing and slowing cancer progression. The nutritional intervention is also beneficial in cancer therapy, including avoiding malnutrition and cachexia and alleviating cancer therapy‐induced symptoms as well as the use of specific diets that may augment concomitant therapies. This review aims to explore these concepts and highlight the need for their integration into medical school curriculum, particularly osteopathic medical education, as nutrition is closely interrelated to the whole‐person patient care approach. These are fundamental concepts that do not gain the recognition they deserve in the typical medical school curriculum, and this may be reflected in clinical outcomes if not appropriately addressed. Addressing this gap in clinical medicine may reduce the risk of cancer, improve patient outcomes, build trust, and decrease the burden of cancer‐related healthcare costs.

## Graphical abstract

A plant‐based diet reduces the risk of cancer by providing essential nutrients and phytochemicals that contain anticancer properties. Osteopathic medicine's holistic approach emphasizes the interconnectedness of body systems and lifestyle factors, including nutrition, to promote overall health and prevent disease. Incorporating nutrition into osteopathic clinical practice for cancer prevention in crucial in supporting health and mitigating cancer risk.

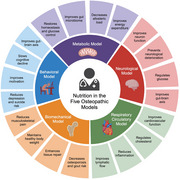

## 1 Introduction

Cancer is a leading cause of morbidity and mortality worldwide. According to the International Agency for Research on Cancer (IARC), approximately 20 million new cases of cancer were diagnosed and 9.7 million deaths were attributed to cancer in 2022 [1]. The American Cancer Society estimates that more than 2 million new cases and 618,120 cancer deaths (approximately 1700 per day) are projected to occur in 2025 [2]. In terms of mortality, lung cancer is the leading cause of death in both males and females, followed by breast or prostate and colorectal cancers [2]. Prostate cancer is the most common cancer in males, followed by lung and colorectal cancers [2]. Breast is the most common in females, also followed by lung and colorectal [2]. Cancer incidence in men peaked in the early 1990s but has been on a steady decline since, whereas the rate has increased slowly since the 1980s in women [2]. Overall, the 5‐year survival rate for all cancer types has increased steadily since the 1970s, likely due to earlier detection and improved treatment modalities [2].

**FIGURE 1:**
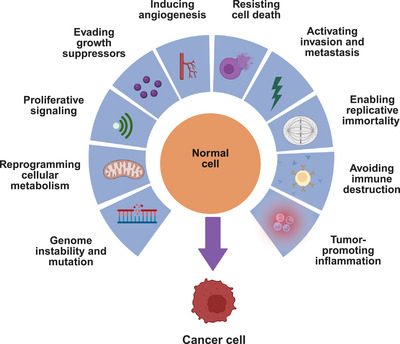
The eight hallmarks of cancer and two enabling characteristics that describe the biological processes underlying malignancy initiation and progression based on the scheme proposed earlier [3, 4].

While cancer development is not understood in its entirety, there are two groups of risk factors: those that are modifiable or lifestyle related, and those that are nonmodifiable or genetic. These risk factors compound with one another over time and increase the susceptibility of normal cells to undergo unregulated cell proliferation, become cancer cells, and lead to tumorigenesis and metastasis [3]. The biology of cancer is described by several hallmarks that allow tumor growth and invasion into surrounding tissue, and they complement one another to enable malignancy to occur [4]. The eight hallmarks of cancer include sustaining proliferative signaling, evading growth suppressors, activating invasion and metastasis, enabling replicative immortality, inducing angiogenesis, resisting cell death, reprogramming cellular metabolism, and avoiding immune destruction [3]. Additionally, two enabling characteristics that are necessary to activate the eight hallmarks involve tumor‐promoting inflammation and genome instability and mutation (Figure 1) [3, 4]. Recent developments have added four emerging hallmarks and enabling characteristics, which consist of unlocking phenotypic plasticity, nonmutational epigenetic reprogramming, polymorphic microbes, and senescent cells [3, 4]. There are specific genes that various individuals are born with that favor the occurrence of these hallmarks, while others are born with normal genes that become mutated due to lifestyle factors, including tobacco use, alcohol consumption, obesity, ultraviolet radiation, viruses, and aging. Specific types of genes are known to cause cancer if inherited with mutations, and these are categorized into tumor suppressor genes, oncogenes, and DNA repair genes. Most cancers are caused by numerous mutations, both germline and acquired, that have built up over time. It is estimated that per 100 cancers, about 5–20 begin from an inherited mutation, suggesting lifestyle and environmental factors are major contributors to cancer development [5]. Therefore, understanding how environmental and lifestyle factors may contribute to acquired mutations is paramount in preventing cancer development and/or slowing down its progression.

In order to reduce the prevalence, progression, and mortality of cancer, it is important to screen to detect any suspicious precancerous or cancerous lesions or traits. Current screening guidelines by the United States Preventive Service Task Force (USPSTF) recommend regular screening for breast, cervical, and colorectal cancers as well as lung cancer in populations at risk. Screening modalities for these types of cancer include mammography (breast), Papanicolaou smear (cervical), colonoscopy (colon), and low‐dose computed tomography (lung). Ovarian, pancreatic, testicular, and thyroid cancer screening have not been shown to reduce cancer‐related mortality or morbidity [6]. According to USPSTF guidelines, bladder, oral, and skin cancer screening does not have sufficient evidence to recommend in asymptomatic individuals [6]. Screening for head and neck cancer should be done as part of the general wellness check, as well as a digital rectal exam and an optional prostate‐specific antigen test in men to screen for prostate cancer. While screening is critical to detect cancer at a preliminary more treatable stage, it is associated with a number of risks, including false positives, false negatives, overdiagnosis, and over testing when a false positive is discovered [7]. These issues can lead to unnecessary invasive procedures that may cause harm to the patient in addition to increased worry, fear, stress, and financial burden [7]. Because many cancers develop slowly over several years as a result of genetic, environmental, and infectious factors and the screening for various cancers may not always be accurate and without risk, it is imperative to adopt prevention as a means to avoid the development of the disease in the first place.

Previous reviews on diet and cancer have only focused on specific dietary patterns, such as the ketogenic or Mediterranean diet, only analyzed the effect of nutrition on a single type of cancer, or only focused on a single dietary component, such as a fruit or vegetable and its anticancer properties [8, 9, 10, 11, 12, 13, 14]. Other reviews have focused on multiple lifestyle factors, such as exercise, rather than solely focusing on nutrition [15, 16]. The most recent review on cancer prevention was published in 2020 and included a broad review of several layers of cancer prevention and did not heavily focus on diet [17]. In this article, we first provide an overview of the ways the standard American diet (SAD) can contribute to cancer development and explain evidence‐based dietary interventions to prevent cancer. Then, we focus on all dietary aspects and their role in preventing various types of cancer as well as the role of diet during cancer therapy. Moreover, the role of nutritional intervention in cancer therapy, including the importance of nutrition in avoiding malnutrition and cachexia and alleviating cancer therapy‐induced symptoms is also discussed. Specific diets that may augment concomitant therapies are described. Additionally, we highlight the educational gap in medical education, particularly as it relates to osteopathic medicine, and practice as well as dietary/nutritional approaches to improve both patient outcomes and their overall quality of life.

## 2 Dietary Contribution to Cancer Development

The SAD, also known as Western Diet, is defined by excess calories from processed foods, added sugars, refined fats and carbohydrates, red meat, and dairy products and a lack of fruits, vegetables, legumes, and whole grains. More specifically, the main components of the SAD include fried foods, omega‐6‐based oils, processed meats (e.g., bacon and deli meat), red meat (e.g., beef, lamb, and pork), refined grains (e.g., white flour and white pasta), and sugar‐sweetened beverages. This diet has unfortunately been adopted by many Americans, and since its beginnings in the late 1940s, it has slowly infiltrated other countries across the globe.

**FIGURE 2:**
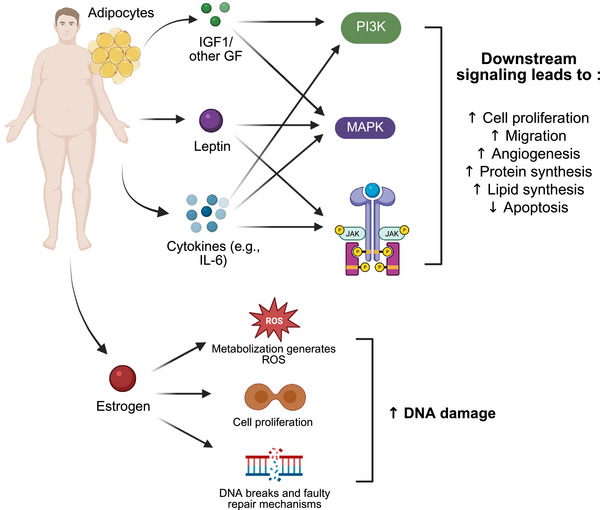
Mechanisms by which obesity promotes cancer development. Ultimately, an environment abundant in adipocytes, estrogens, and other inflammatory molecules promotes uncontrolled cell proliferation and induces DNA damage. *Abbreviations*: DNA, deoxyribonucleic acid; IGF‐1, insulin‐like growth factor 1; IL‐6, interleukin‐6; JAK, Janus kinase; MAPK, mitogen‐activated protein kinase; PI3K, phosphoinositide 3‐kinases; ROS, reactive oxygen species.

The SAD diet promotes cancer development and progression in many ways. It promotes obesity, which, in turn, has been linked to numerous types of cancer, including breast, uterine, kidney, esophageal, gallbladder, pancreatic, liver, and colorectal cancer [18]. Recently, Shi et al. [19] conducted a meta‐analysis of the effect of body mass index (BMI) and cancer risk and concluded that BMIs falling in the overweight and obese categories were associated with an increased risk of several types of cancers compared with the normal or underweight categories, and a weight gain greater than 5 kg was associated with an increased risk of overall cancer. A recent prospective clinical trial demonstrated that bariatric surgery in women with obesity resulted in a significantly reduced risk of breast cancer, underscoring the pivotal role that obesity may play in especially hormone‐specific cancer types [20]. The mechanisms relating obesity to cancer risk are multifaceted and still under investigation, but mostly proposed to relate to the proinflammatory environment that adipocytes propagate and the hormone imbalance that results [21]. Several common ways obesity may promote cancer initiation and progression are illustrated in Figure 2.

**FIGURE 3:**
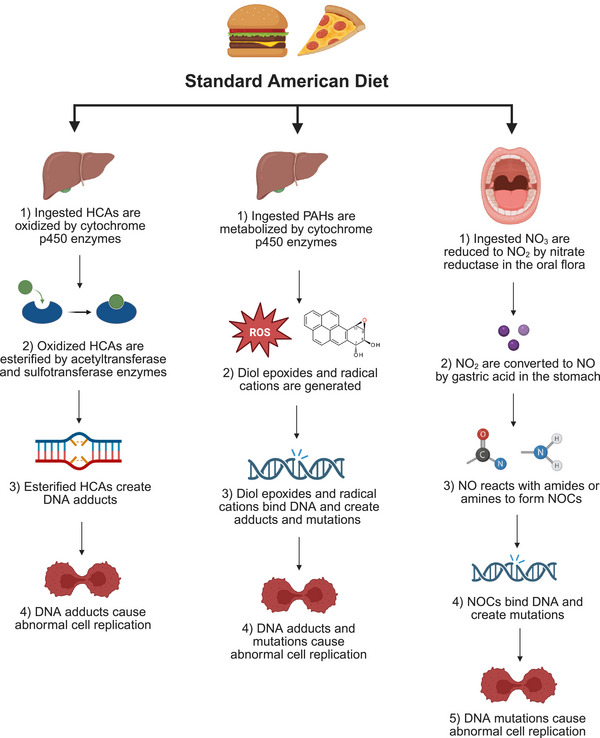
Various mechanisms by which the standard American diet promotes cancer development. Frying, grilling, smoking, and cooking meats and other foods at high temperatures generates carcinogens, such as HCAs, PAHs, and acrylamides. These cause DNA adducts, mutations, and lead to uncontrolled cell proliferation. Processed meats (sausage, ham, bacon, and hot dogs) contain preservatives, such as nitrates and nitrites that are converted to carcinogenic NOCs. These bind DNA and create instability, causing DNA mutations and abnormal cell division. DNA, deoxyribonucleic acid; HCAs, heterocyclic amines; NO, nitric oxide; NO_2_, nitrite; NO_3_, nitrate; NOCs, *N*‐nitroso compounds; PAHs, polycyclic aromatic hydrocarbons; ROS, reactive oxygen species.

In addition to causing obesity, popular foods in the SAD have numerous known substances that increase the risk of developing cancer, or carcinogens, and are traditionally prepared using methods that promote the formation of various carcinogenic compounds. Heterocyclic amines (HCAs) are mutagenic compounds produced in meats cooked at high temperatures for prolonged periods of time. They are oxidized by cytochrome P450 enzymes and converted to ester forms by acetyltransferase and sulfotransferase, which then produce DNA adducts that lead to abnormal cell replication (Figure 3) [22]. In the last few decades, many epidemiological studies have investigated the association between HCA exposure and cancer risk and reported a high intake of well‐cooked meats may be associated with an increased risk of colorectal, esophageal, stomach, lung, pancreas, prostate, and breast cancer. Many of these studies have a small sample size and statistical power, but the findings are consistent with in vitro and in vivo data showing similar results [23].

Polycyclic aromatic hydrocarbons (PAHs) are another group of well‐characterized compounds that are carcinogenic, and humans are exposed to them environmentally (via vehicle exhaust emissions, cigarette smoke, and forest fires) and from dietary sources, although the latter is the major means of exposure in nonsmokers [24]. PAHs are generated when high temperatures, organic matter, and low oxygen levels are present and lead to incomplete combustion (Figure 3). If food is involved, the PAHs generated infuse into the product and are consumed in the diet. Foods containing high levels of lipids (fish, beef, and poultry) are major deliverers of PAHs into the gastrointestinal system because they are lipophilic in nature and bind to the cell membrane. Roasting, frying, smoking, and grilling are known to produce PAHs in larger amounts than alternative cooking methods, and this is true of various foods, including vegetable oils, cheeses, and grains, not just meats [25].

Nitrates and nitrites are found naturally in green leafy vegetables, but are also used as preservatives for meats, such as ham, bacon, and hot dogs and in curing salts for red meat. When ingested, they can react with amines and amides to form N‐nitroso compounds (NOCs), which are known carcinogens in experimental animals and likely in humans (Figure 3). Many vegetables contain vitamin C and E as well as other phytochemicals that inhibit the formation of the carcinogenic NOCs, while heme in red meat stimulates NOC formation [26, 27]. Nitrates and nitrites from processed and cured meats have been associated an increased risk of colorectal cancer, prostate cancer, thyroid cancer in men, ovarian, gastric, esophageal, bladder, and pancreatic cancers [28]. In fact, the World Health Organization released a public health statement in 2015 classifying processed meats (hot dogs, ham, sausages, corned beef, biltong, beef jerky, and canned meat) as Group 1 carcinogens, meaning sufficient evidence has shown human consumption of these food items causes colorectal cancer. This statement also declared that red meat is probably carcinogenic to humans and classified it as a Group 2A carcinogen, meaning there is limited evidence that it causes cancer, but strong mechanistic evidence suggests it may do so [29]. These recommendations were made based upon the IARC Working Group's evaluation of over 800 research studies that investigated many types of cancer and associations with red and processed meat in various countries and diet styles. The conclusions of the IARC Working Group was based on 20 of the 800 studies, and they stated that the Working Group were identifying hazard only, not cancer risk [30]. They emphasized that red meat does have nutritional value, serving as a good source of iron, zinc, and B vitamins, and risk assessments should be conducted to assess the advantages and disadvantages of limiting meat intake as a public health concern [29].

Acrylamide is another by‐product of cooking that is considered a Group 2A carcinogen, and it is produced during the Maillard reaction when glucose or fructose reacts with asparagine [31]. This causes the color change and browning of food during frying, baking, or roasting, and the levels of acrylamide accumulated in foods depends on several factors, including the baking temperature, cooking time, and the quantity of natural asparagine and reducing sugar in the food. Foods highest in acrylamide concentration are potato chips, French fries, pan‐fried potatoes, crackers, crisp breads, breakfast cereals, corn chips, and soft breads [32]. Although in vitro and in vivo evidence have shown acrylamide to cause cancer, epidemiological studies have found inconsistent results and cannot conclude it is a known cause of cancer in humans [33, 34]. Further studies should aim to use longitudinal cohorts and measure their acrylamide exposures over time in order to determine whether acrylamide in the diet increases cancer risk.

Overall, although the link between the SAD and cancer risk has been a growing area of research, many questions remain unanswered. However, public health officials and other health professionals must err on the side of caution when making recommendations concerning dietary cancer prevention. While data may be inconclusive regarding certain foods, the public should be aware of how carcinogens in the diet may play a role in cancer development and progression, and measures should be taken to avoid generating these carcinogens when possible. However, restricting too many foods may lead to many negative outcomes, including decreased food enjoyment and anxiety among consumers. Additionally, care should be taken to avoid self‐blame among cancer patients regarding their food choices, but these factors should not negate the need for proper nutrition counseling. Because obesity may play a role in carcinogenesis, it is crucial to encourage weight maintenance and control to prevent cancer as well as many other chronic diseases, including diabetes and cardiovascular disease. Large prospective studies are necessary to provide a better understanding of how the SAD, cooking methods, and other dietary components may contribute to cancer risk so that public health recommendations can be modified and updated accordingly.

## 3 Evidence‐Based Dietary Interventions for Cancer Prevention

While it is important to understand the ways the SAD diet can contribute to cancer development, it is equally of importance to be informed on the many protective foods we can include in the diet that may reduce the risk of cancer. This section aims to explore evidence‐based dietary interventions for cancer prevention, focusing on specific foods and ingredients that display cancer‐protective properties that may reduce cancer risk. Furthermore, this section provides a discussion of certain dietary patterns, including vegan, ketogenic, and Mediterranean diets, that may play a role in cancer prevention.

### 3.1 Optimal Anticancer Diet

Research on cancer risk reduction with nutritional science has been limited due to the fact that nutrition is difficult to research because of participant variation (sex, race/ethnicity, economic status, and fitness), recall bias, and other errors associated with collecting dietary data [35]. Previously, most nutritional research on cancer prevention was reductionist in design and only focused on single nutrients or components of the diet without assessing the complexity of food synergism in carcinogenesis. In the past few decades, studies have begun to investigate overall dietary patterns over longer periods of time and their effects on cancer incidence and mortality [36].

In general, health‐promoting diets are predominantly comprised of plant‐based foods, such as fruits, vegetables, whole grains, legumes, spices, nuts, and seeds. These foods contain a plethora of vitamins, minerals, and plant secondary metabolites (phytochemicals) that prevent numerous diseases, including cancer. For example, various fresh fruits and dried fruits, including pomegranate, mango, banana, papaya, guava, avocado, mangosteen, and litchi, as well as vegetables and spices, particularly garlic and ginger, have displayed cancer preventive properties across multiple cancer types [37, 38, 39, 40, 41, 42, 43, 44, 45, 46, 47, 48, 49]. The phytochemicals found in plant‐based foods target multiple cell‐signaling pathways that are involved in tumorigenesis [50, 51, 52, 53, 54]. Notably, carotenoids, flavonoids, organosulfur compounds, phenolic acids, phytosterols, stilbenes, and other phytochemicals have demonstrated potent anticancer activity in many types of cancers (Table 1) [55, 56, 57, 58, 59, 60, 61, 62, 63, 64, 65, 66, 67, 68]. In particular, organosulfur compounds, such as isothiocyanates and sulforaphane, are abundant in cruciferous vegetables (broccoli and cabbage) and have shown to induce apoptosis, cell cycle arrest, reduce angiogenesis, and influence gene expression of various oncogenes and tumor suppressor genes [69, 70, 71]. Whole grains contain vitamin E, lignans, fiber, and phytic acids, all of which have demonstrated anticancer properties in previous studies [72]. Teas and various spices also contain numerous phytochemicals, including, catechins, curcumin, diallyl disulfide, capsaicin, gingerol, anethole, diosgenin, and eugenol, that display cancer‐protective properties [52, 73, 74, 75]. The molecular mechanisms by which these phytochemicals and plant compounds exert their anticancer effects is a growing area of nutritional science, and future research in this area may pave the way for future cancer therapeutics [36, 76, 77]. It is important to note that many plant foods contain compounds naturally and are treated with pesticides that are known rodent carcinogens, but the implications of these in human cancers is not completely elucidated. It is likely that the many anticarcinogenic vitamins and antioxidants abundant in fruits and vegetables contribute to the lower cancer rates associated with plant‐based diets despite the use of pesticides [78]. While clinical studies investigating plant‐based diets on nutrition are lacking, there are many observational studies, including longitudinal cohort studies and case‐reports that show a decreased risk of overall cancer risk with predominantly plant‐based and Mediterranean diets [79, 80, 81]. Additionally, plant‐based diets may offer protection after a cancer diagnosis has been established, improving overall cancer prognosis, particularly in breast, colorectal, and prostate cancers [80, 81, 82, 83].

As nutrition has become an interest in anticancer prevention and adjunctive therapy, nutritional supplements for cancer prevention have also been on the rise. According to the 2023 Council for Responsible Nutrition Consumer Survey on Dietary Supplements, over half of all Americans are taking dietary supplements and 55% qualify as “regular users” [84]. Dietary supplements have become popular because they are an easy way to fill in nutritional shortcomings and are widely accessible. However, instead of serving as a supplemental add‐on to the diet to meet nutritional needs, they have become a replacement for eating foods high in nutrients. An even larger public issue is the lack of regulation in what compounds constitute these “cancer‐fighting” supplements as well as the marketing schemes supplement companies advertise to the public, particularly to susceptible populations, such as cancer patients, who are looking for any possible remedy to reduce their disease burden. These companies capitalize on prior research showing phytochemicals and bioactive compounds in natural foods demonstrate anticancer effects, but they fail to recognize that a lot of good things may not always be better and may, in fact, cause harm. Furthermore, expert committees have analyzed the current evidence and clinical trials and determined that nutritional supplements have little to no benefit in preventing cancer [85].

Theoretically, the optimal anticancer diet would consist of a wide variety of fruits, vegetables, whole grains, spices, and herbal teas prepared without frying, roasting, smoking, or grilling to avoid the production of carcinogens discussed in the prior section. Eating a diet with a variety of these plant‐based foods would provide the body with numerous phytochemicals that may prevent cancer at various points of its initiation and progression. Currently, there are no evidence‐based recommendations for nutritional supplement use for cancer prevention. The Recommended Dietary Allowances and Dietary Reference Intakes outline the recommendations for essential nutrients, but there are no established guidelines for the recommended intake for bioactives found in natural foods [86, 87]. Additional studies should aim to investigate the use of supplements for cancer prevention and intervention, formulate novel biotechnologies to deliver these supplements to optimize bioavailability, and explore toxicities of bioactives to avoid potential harm. In the meantime, legislation regarding supplement formulations and advertisements should be modified for public health concerns.

### 3.2 Dietary Patterns

Specific dietary regimens are a growing area of interest in cancer prevention. Several dietary patterns, such as the vegan, ketogenic, and Mediterranean diets, have demonstrated promising anticancer effects [36, 37].

Veganism excludes meat, poultry, dairy, eggs, fish, and other animal products from the diet. While high in the various phytochemicals described earlier, the vegan diet may be low in vitamin D, vitamin B12, calcium, iodine, and overall calories. The vegan diet is associated with decreased risk of numerous chronic diseases, including cancer, but most of the evidence is in vitro and in vivo and may not be relevant to humans. The vegan diet creates an environment in which cancer would not be favored due to the abundance of cancer protective phytochemicals, vitamins, and antioxidants; deficiency in overall calories which would avoid obesity; and the avoidance of meats and processed meats, which may be carcinogenic. Arguably, there can be a lot of variation in what makes up the foundation of a vegan diet, that is a vegan diet comprised of fried foods, processed grains, and high‐fat foods is not the same as a plant‐based vegan diet comprised of fruits, vegetables, whole grains, legumes, nuts and seeds. This discrepancy is believed to be the reason various studies come to conclusions about vegetarian and vegan diets and cancer risk that appear to conflict [88]. Many large prospective observational studies have found a 10–12% reduction in overall cancer risk in meat‐free diets, which includes both vegetarian and vegan diets [89]. A recent meta‐analysis found that a vegetarian diet offers a significant protective effect on total cancer incidence, reducing risk by 8%, and a vegan diet conferred up to a 15% risk reduction of incidence of total cancer [90]. Nonetheless, additional clinical studies evaluating plant‐based vegan diets and overall cancer risk are necessary.

The ketogenic diet is a high fat, low carbohydrate diet and has been recently promoted for weight loss as well as reducing risk of diabetes and cancer. The goal of ketogenesis is to decrease glucose to the point that the primary energy supply to the body becomes ketone bodies, and these are produced when adipose tissue is broken down by fatty acid oxidation. These ketone bodies are converted to acetyl coenzyme A to enter the citric acid cycle to produce energy [91]. The Warburg effect describes the way in which cancer metabolism differs from noncancerous cell metabolism [92]. Cancer cells rely on aerobic glycolysis, whereas normal cells generate energy mainly through mitochondrial oxidative phosphorylation. This phenomenon underlies the potential mechanisms that a ketogenic diet may decrease cancer risk, because in a ketogenic environment, normal cells are able to use ketone bodies for energy while cancer cells cannot and are deprived from an energy source. There are several studies showing promising anticancer effects from a ketogenic diet on prostate, gastric, neuroblastoma, and lung cancers in vitro, and there are several in vivo studies suggesting a ketogenic diet decreased neoplastic growth; however, there is an overall lack of clinical studies to demonstrate these effects in humans [93, 94, 95]. The major drawbacks of the ketogenic diet in cancer patients are mainly weight loss, especially in a patient population that is at risk of cachexia, as well as hypoglycemia, nausea, vomiting, and lethargy which predominantly occur when entering a state of ketosis. Additionally, bone loss and hypercholesterolemia with subsequent renal damage may occur due to the limitation of carbohydrates and emphasis on high dietary fat intake [36]. Further analysis on the use of ketogenic diets with concomitant cancer therapy is discussed in the next section.

In summary, the risks and benefits regarding various dietary patterns for cancer prevention should be addressed thoroughly before recommending to patients. Further research in this area should be performed to provide a better understanding of the ways dietary patterns can influence specific types of cancer and as well as overall cancer risk.

## 4 The Impact of Nutrition on Cancer Prognosis and Cancer Therapy

Whilst several diet‐based approaches for cancer prevention have been established, a discussion on how we can best support patients previously diagnosed with cancer by optimizing nutrition is warranted. An abundance of evidence has shown that the role of nutrition in cancer therapy is largely important not only for patient quality of life and functionality, but also for longevity and clinical prognosis and survival. Nutrition is a crucial component of patient care that is often overlooked, and it's use in oncologic medicine is largely inconsistent [96]. This section aims to address the role of nutrition in combating cancer cachexia and malnutrition, the ways diet can alleviate cancer therapy‐induced symptoms, and specific diets that may benefit concomitant cancer therapies to improve cancer prognosis (Table 2).

**TABLE 1:** Phytochemicals found in plant‐based diets with potential anticancer effects.

Phytochemical classes	Examples	Food sources	Effects on cancer hallmarks	Molecular mechanisms	References
Alkaloids	Caffeine, theobromine, theophylline, tomatine, solanine	Coffee, cacao, herbal teas, tomatoes, and potatoes	↑Apoptosis	↑Autophagy; ↑ferroptosis; ↑ROS	[29, 32, 33, 37, 38, 39, 64]
Phenolics	Curcumin, quercetin, epigallocatechin gallate, resveratrol, piceatannol, hydroxybenzoic acid, hydrocinnamic acid, isoflavones, and anthocyanins	Coffee, oranges, berries, chestnuts, cabbage, olive oil, and grapes	↑Apoptosis; ↓cell proliferation; ↓inflammation	↓Cellular damage; ↓ROS; ↓VEGF; ↓fatty acid synthase activity; ↓tyrosine kinase activity; ↓nitroso compound formation; ↑AMPK; ↑Bax; ↑Bak; ↑p21; ↓COX‐2; ↓PGE‐2; ↓Bcl‐2; ↓Bcl‐xL; ↓NF‐κB	[26, 27, 28, 29, 30, 31, 32, 34, 35, 37, 38, 39, 65, 66, 67, 68, 69, 70, 71]
Sulfur compounds	Isothiocyanates, sulforaphane, indoles, and allylic sulfur compounds	Broccoli, cabbage, and Brussels sprouts	↑Apoptosis; ↓angiogenesis	↑G2/M cell cycle arrest; ↑p21; ↑Bax; ↓cyclin B1; ↓cyclin D; ↓VEGF; ↓HIF‐1α	[33, 35, 37, 38, 39, 46, 72]
Terpenoids	β‐Carotene, lycopene, phytosterols, lutein, ursolic acid, and canthaxanthin	Tomatoes, oranges, carrots, bell peppers, almonds, and wheat germ	↑Apoptosis; ↓cell proliferation; ↓cell invasion; ↓cell adhesion; ↑immune response	↑G1/G0 cell cycle arrest; ↓AMPK; ↓HIF‐1α; ↓VEGF; ↑sphingomyelin cycle; ↑ceramide generation; ↓cholesterol synthesis	[28, 30, 31, 37, 38, 39, 73]

*Symbols and abbreviations*: ↑, increase or upregulation; ↓, decrease or downregulation; AMPK, adenosine monophosphate activated protein kinase; Bak, apoptosis‐related BCL2 antagonist/killer 1; Bax, Bcl‐2‐associated X‐protein; Bcl‐2, B‐cell lymphoma 2; Bcl‐xL, B‐cell lymphoma‐extra‐large; COX‐2, cyclooxygenase 2; HIF‐1α, hypoxia‐inducible factor 1α; NF‐κB, nuclear factor‐κB; PGE2, prostaglandin E2; ROS, reactive oxygen species; VEGF, vascular endothelial growth factor.

**TABLE 2:** Summary of the impact of nutrition on cancer therapy.

Nutritional strategies in oncology	Evidence‐based approaches and current state of knowledge
Combating cachexia and malnutrition	Pretreatment malnutrition, cachexia, and sarcopenia are major determinants of treatment tolerance and clinical outcomes [98, 99, 100]. Studies have shown that nutritional intervention in cancer patients prior to undergoing therapy can improve quality of life and overall survival [104, 107, 108]. There is not consistent malnutrition screening for cancer patients [106]. There is a need for nutritional screening and evidence‐based recommendations for dietary guidance prior to initiating treatment, particularly in the elderly.
Alleviating cancer therapy‐induced symptoms	Many patients undergoing cancer therapy experience many adverse effects that influence their dietary habits [109]. These adverse effects include stomatitis, xerostomia, diarrhea, nausea, vomiting, dysphagia, dysgeusia, and dysosmia, resulting in reduced efficacy [109]. Vitamin/other dietary deficiencies may be exacerbated due to cancer therapeutic agents [109, 110, 111]. Emerging research on the miracle berry [112, 113], guarana fruit and cat's claw supplementation may mitigate chemotherapy induced symptoms [114, 115, 116, 117, 118, 119]. Personalized dietary plans should be implemented in these populations to increase adherence to treatment plans and quality of life [111].
Diets that may improve clinical outcomes	Intermittent fasting may improve clinical symptoms according to recent in vivo and case studies [121, 122, 123, 124, 125, 126, 127, 128, 129, 130, 131]. A recent clinical trial found that a fasting mimicking diet may be used in combination with standard cancer therapies to boost the immune system and reprogram metabolic parameters that may influence cancer progression [126]. Dietary patterns influence the types of gut microbiota that are abundant, and these microbiotas may influence the effectiveness of standard cancer treatments [81, 131, 132, 133, 134, 135, 136, 137, 138]. Overall, there is a lack of clinical studies investigating the effects of specific diets on cancer patients undergoing cancer therapies.

### 4.1 Combating Cachexia and Malnutrition With Nutritional Intervention

Cachexia is a complex metabolic disease that is a known contributor to functional decline in the cancer patient population, and it is defined by progressive skeletal muscle mass loss with or without fat mass loss. More specifically, the diagnostic criteria include the loss of 5% or more of weight loss or 2% or more in patients who are already underweight according to their BMI [97]. There are several known contributors to cachexia that are due to pathophysiologic processes that occur during cancer progression that lead to loss of appetite, increased protein catabolism, increased resting energy expenditure, and the promotion of a systemic inflammatory environment [98]. During times of prolonged fasting in normal subjects, there are protein‐sparing metabolic adaptations that increase autophagy, while suppressing anabolic pathways, causing minimal muscle loss. However, in a state of inflammation seen in chronic disease states, such as cancer, these adaptations are disrupted, and protein and muscle loss are much higher [99]. The degree of skeletal muscle loss, or sarcopenia, observed as cancer progresses has been shown to be associated with increased mortality. A recent study showed that preimmunotherapy sarcopenia was associated with poor outcomes in patients with non‐small cell lung cancer receiving cytotoxic drug treatment [100]. Sarcopenia is especially evident among the elder population, of whom often have a lower baseline level of skeletal muscle mass, increased muscle attenuation, and a lower BMI [101]. This population is also prone to age‐related anorexia which may worsen malnutrition particularly in the setting of cancer. Unfortunately, there is a lack in research studies designed to evaluate nutritional interventions in older adults with cancer, which restricts evidence‐based recommendations for augmenting nutrition practices in this population [102]. Consequently, trials designed to investigate nutritional interventions in the elderly diagnosed with cancer are merited to better provide nutritional guidance to this group of patients.

Malnutrition and cachexia are common problems also among those with head and neck cancer, where not only do patients have the metabolic derangements favoring anorexia, but also mechanical obstacles from carcinogenic tissue [103]. Previous research studies have shown that nutritional and physical prehabilitation intervention have a beneficial effect on the nutritional status and overall outcome of patients with head and neck cancers [104]. A recent systematic review concluded that no clear statement could be made regarding the effectiveness of intensive nutritional therapy in patients with head and neck cancer treated with radiotherapy or chemoradiotherapy [105]. The lack of consistency among studies and high risk of bias led to their conclusion, and the authors highlight the need for high quality studies on this topic [105].

There have been initiatives to screen cancer patients at risk of malnutrition and cachexia on a global scale, yet this remains a component of oncologic medicine that is not consistent among healthcare institutions [106]. Screening for malnutrition and cachexia in newly diagnosed cancer patients should be routine in order optimize patient nutrition, especially prior to neoadjuvant or adjuvant treatment which may worsen nutritional status. Previous studies have shown that nutritional intervention in cancer patients prior to undergoing neoadjuvant therapy can improve the quality of life during treatment and overall survival as well as improve operative outcomes in surgical patients [107]. A recent review concluded that specifically high‐protein supplementation in patients undergoing cancer therapy improves muscle strength, lessens weight loss, and decreases hospitalization rates and may be a useful adjunct to oncologic practice [108]. Therefore, due to the negative impact of malnutrition and cancer cachexia on clinical outcomes and quality of life, standardized malnutrition screenings should be a routine part of cancer care and individualized high‐protein dietary plans should be constructed to optimize patient health prior to and during cancer therapies.

### 4.2 Alleviating Cancer Therapy‐Induced Symptoms With Nutrition

While cancer therapies have become extremely advanced over the past decade and have improved cancer‐associated morbidity and mortality, the side effects and metabolic alterations that patients undergo while on these therapies may be detrimental to their quality of life. Various conditions patients face on these regimens include fatigue, stomatitis, xerostomia, diarrhea, nausea, vomiting, dysphagia, dysgeusia, and dysosmia [109]. For example, multikinase inhibitors used in the treatment of advanced thyroid cancer are extremely efficacious, but may reduce the quality of life of patients due to drug‐induced nausea, anorexia, stomatitis, and diarrhea [110]. These adverse effects often lead to intolerance and require decreasing the medication dosage or discontinuing it altogether, ultimately causing reduced efficacy [110]. A lack of nutritional consideration in these patients can adversely affect prognosis and interfere with their capacity to adhere to proper therapies. Detecting dietary deficiencies early in the treatment plan is crucial, and missing these deficiencies can cause a substantial decline in nutritional status and diminish patient survival. This is particularly relevant in certain cancer types that commonly present with malnutrition and anorexia at the time of diagnosis, such as pancreatic cancer, putting them at significant risk for nutritional decline [111].

Several dietary approaches to mitigate cancer‐related symptoms or chemotherapy‐related adverse effects have been proposed. There is an area of emerging research regarding the use of the miracle berry (*Synsepalum dulcificum*, an indigenous fruit of tropical West Africa) supplementation in cancer supportive care to combat chemotherapy‐ and radiation‐induced dysgeusia, malnutrition, and taste dysfunction. Several studies have shown that this medicinal plant contains a glycoprotein, namely miraculin, which acts on taste receptors to modify acidic and sour tastes, and this may be beneficial in patients undergoing cancer therapy who are experiencing dysgeusia, malnutrition, and taste dysfunction [112, 113]. Moreover, several preliminary clinical studies have showed that dietary supplements developed using guaraná fruit (*Paullinia cupana*) and cat's claw (*Uncaria tomentosa*), both from the Amazon region, may successfully manage cancer treatment‐related side effects, such as neutropenia, anorexia and fatigue and improve overall quality of life in cancer patients [114, 115, 116, 117, 118, 119]. Additional studies should aim to investigate the use of these dietary agents in cancer therapeutics to better help alleviate symptoms of dysgeusia and malnutrition in this population to improve clinical outcomes and prognosis.

Stomatitis and xerostomia, which may be side effects from radiation, anthracyclines, or other cytotoxic drugs, can worsen malnutrition in cancer patients by making it very difficult and painful to swallow foods. In these cases, personalized dietary plans that focus on soft foods, blended or soaked foods that are moisturized, highly calorie‐dense foods, and the use of straws. Patients with cancer therapy‐induced diarrhea may need small frequent meals that are high in soluble fiber, and those with chemo‐ or radiation‐induced nausea and vomiting may benefit from small frequent meals that consist of bland and odorless foods [111]. In both cancer therapy‐induced diarrhea and nausea and vomiting, electrolyte disturbances must be considered, and diets must be modified to maintain adequate electrolyte balances.

The evidence suggesting the use of probiotics in cancer therapy‐induced symptoms is contradictory. A recent review found that probiotics and synbiotics have no significant effects on reducing chemotherapy‐associated toxicity and diarrhea in colorectal cancer patients; however, very few studies were included in the review [120]. Further research on this topic is needed to better elucidate the need for this type of supplementation in colorectal cancer and other cancer patients.

Because patient adherence to treatment regimens is necessary to combat metastatic growth, it is of utmost importance that we learn to mitigate adverse effects associated with cancer therapies to increase patient compliance. Early detection of malnutrition and nutritional deficiencies that result from stomatitis, xerostomia, diarrhea, and other adverse effects from cytotoxic therapies and medications is crucial. Addressing these challenges early in the treatment plan and providing dietary guidance to best support patients dealing with cancer therapy‐induced symptoms may prevent malnutrition‐related complications.

### 4.3 Diets That May Enhance and Improve Clinical Outcomes in Patients Undergoing Cancer Therapies

Previously, we outlined the various dietary patterns that may prevent cancer initiation and progression. However, a discussion of dietary patterns and their anticancer effects on patients already undergoing cancer therapy is warranted. This section aims to describe emerging literature using various dietary patterns, such as intermittent fasting, as an adjunctive therapy during cancer treatment.

Intermittent fasting requires the complete withholding of calorie intake for a specified time, and this dietary pattern has been studied for decades in chronic diseases. In cancer studies particularly, intermittent fasting has been shown to be a useful adjunct to chemotherapy in in vivo models with breast, neuroblastoma, pancreatic, and colorectal cancers as well as melanoma [121]. The proposed underlying mechanism for intermittent fasting in cancer prevention is similar to ketogenesis, but with a concentration on switching from anabolic processes to resource maintenance. This ultimately leads to decreased gene expression of anabolic proteins, such as growth factors. Specifically, this dietary pattern has been shown to decrease insulin‐like growth factor‐1, which reduces the activation of the rat sarcoma/mitogen‐activated protein kinases and phosphoinositide 3‐kinase pathways, and activates adenosine monophosphate‐activated protein kinases (AMPK), all of which lead to inhibited cell proliferation and autophagy induction [122, 123, 124]. A case study previously demonstrated that intermittent fasting both before and after chemotherapy improved clinical outcomes, and this is in congruence with in vivo data [125]. Another study found that patients who used intermittent fasting while undergoing chemotherapy reported significantly less side effects, such as nausea, weakness, and fatigue, which may limit the amount or duration of chemotherapy treatment [125]. While these studies are promising, intuitively, the major risk in using intermittent fasting for cancer is malnutrition and weight loss. Further studies must be aware of this fact and may opt for reduced fasting time periods to avoid this serious risk.

A clinical trial investigated the safety and anticancer effects of a 5‐day fasting‐mimicking diet during cancer therapies [126]. The trial included 101 patients with various types of cancers, and dietary intervention consisted of a 5‐day fasting mimicking diet followed by 16–23 days of refeeding. This cycle was repeated various times depending on concomitant cancer therapy scheduling, but the mean cycles among the observed patients was four. This dietary pattern was determined to be safe and resulted in anticancer metabolic changes as well as reshaping anticancer immunity, as evidenced by reduced blood glucose, BMI, downregulated immunosuppressive myeloid cells, increased cytotoxic cells, and enhanced T‐cell activation. These findings suggest that a fasting mimicking diet may be used in combination with standard cancer therapies to boost the immune system and reprogram metabolic parameters that may influence cancer progression [126]. Additional clinical trials are needed to better understand the role of fasting as a complimentary treatment when paired with cancer therapies.

A recent systematic review investigated the associations between diet and the effectiveness of lung cancer treatment. Twenty articles, including randomized controlled trials, cohort, and observational studies were included and the authors concluded that a supportive diet rich in phytonutrients and antioxidants should be the standard of care in the treatment of patients undergoing lung cancer therapy [127]. More specifically, increased protein and energy intake was associated with improved life quality, function, strength, and symptoms in patients with lung cancer undergoing therapy, and particularly the intake of omega‐3 fatty acid had some inflammatory‐regulating effect in lung cancer patients undergoing chemotherapy and radiotherapy [127]. Another review explored the effects of dietary interventions and patterns and the effect on neuroendocrine neoplasms (NEN) and determined that nutritional management in NEN treatment optimizes patient outcomes and significantly improves patient quality of life and survival [128, 129]. Intermittent fasting may improve tumor response to NEN cancer therapies due to its inflammatory‐combating weight control effects, as well as improve circadian rhythms which may augment cancer treatments [81, 130, 131]. However, the authors point out that intermittent fasting may exacerbate nutritional deficiencies that may already be present, such as niacin or vitamin D deficiencies, and this could worsen clinical outcomes particularly in patients with NEN who are already at risk for these deficiencies.

Another recent systematic review found that a higher intake of plant‐based foods improved the prognosis in cancer survivors [132]. Specifically, a high intake of whole grains and fiber was associated with improved prognosis in both colorectal and breast cancer patients. A high intake of vegetables was associated with improved prognosis in prostate cancer patients, and a high intake of fruit and moderate intake of soy products was associated with improved prognosis in breast cancer patients. The role of plant‐based diets in cancer patients undergoing treatment is still uncertain, but this review suggests that a plant‐based diet high in fruits, vegetables, whole grains, and moderate soy consumption may be beneficial to clinical outcomes [132].

Finally, there is emerging evidence on the role of the microbiome and the effectiveness of cancer therapy [133, 134, 135, 136, 137, 138]. Certain dietary patterns influence the types of gut microbiota that are abundant, and these microbiotas may influence the effectiveness of standard cancer treatments [139]. This is a developing field of research, and more investigation is needed in this area to better elucidate the effects of dietary patterns on the microbiome and the subsequent impact on chemotherapy and radiotherapy response in various cancer types.

## 5 Addressing the Gap in Medical Education and Clinical Practice

As previously discussed, what we do and do not put on our plates may have a profound impact on our cancer risk stratification. Nutrition in the context of clinical medicine is crucial for primary prevention of many chronic diseases, including cancer. Unfortunately, nutrition is not a foundational science taught in the US medical schools. This is illustrated by recent reports suggesting that over half of medical graduates agree that their nutrition education in medical school is subpar, and they would not feel comfortable making suggestions to patients [141, 142]. A 2020 study showed that US medical students on average receive 19.6 h of nutritional education during their entire time in medical school, which does not meet the minimum of 25 h recommended by the National Academy of Sciences [142]. Additionally, 80% of the nutrition education in medical schools is added to the curriculum mainly as an aside; that is, instead of nutrition serving as a stand‐alone course, it is more of an add‐on to other designated courses. If at all, nutrition education is presented to students in their preclinical years [142]. While learning the science behind nutrition and chronic disease prevention is important, this curriculum design may not be the most advantageous. Two critical parts of nutrition, that is, motivational interviewing and patient‐centered communication, are more important skills learned during clinical years. Perhaps witnessing the impact of nutrition on medical treatment and patient outcomes during the clinical years of medical school would provide students with a more comprehensive understanding of why it is important in clinical practice. Furthermore, it is important for medical professionals to be educated about nutrition not only to provide informed care to their patients, but also to support their own physical health and mental wellbeing to sustain the demanding nature of their work. Given that the chronic disease epidemic has been on the rise for several decades now, nutrition is not given the emphasis it deserves in medical education as a means of primary prevention. Sufficient nutrition education for all healthcare workers, especially future physicians, is necessary to ensure the healthcare team works effectively to support patients’ needs and improve patient outcomes.

Osteopathic medicine is a growing branch of healthcare that incorporates the traditional 4 years of medical school with additional training in osteopathic manipulative medicine, which involves hands‐on training techniques to treat and prevent illness. This form of medicine was established by Andrew Taylor Still toward the end of the 19th century [143]. Osteopathic principles blended traditional Western medicine with Native American healing philosophies and shifted the focus from treating diseases to promoting overall health and wellbeing. Today, it is estimated that there are 200, 000 clinicians worldwide in 46 countries that practice osteopathic medicine [143]. Osteopathic medicine emphasizes a holistic approach to healthcare, focusing on the interconnectedness of the body's systems and importance of disease prevention. Nutrition is a cornerstone of this approach because it impacts the body's inherent ability to maintain health and prevent diseases, such as cancer. Several core principles of osteopathic medicine include the following: the body is a unit as a whole, interconnected system; the body has an innate ability to self‐heal and self‐regulate; structure and function are interrelated; and osteopathic physicians seek to address root causes of illness and not just address the symptoms of disease [144].

**FIGURE 4:**
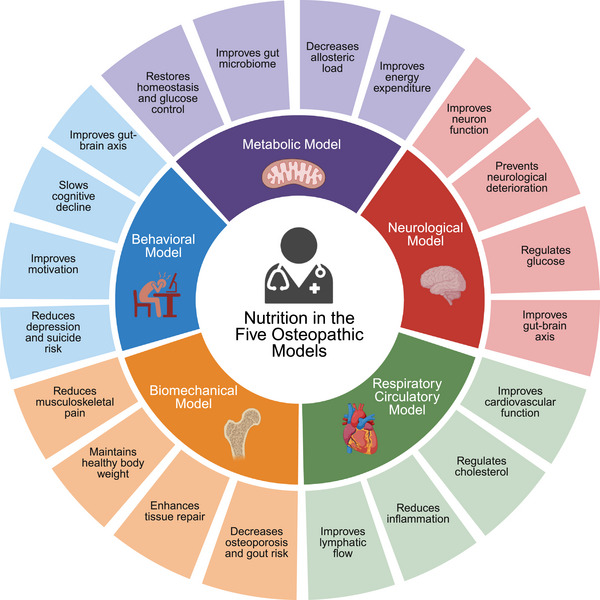
The role of nutrition in each of the five physiologic models of osteopathic medicine. Proper nutrition may enhance the body's inherent ability to heal and maintain health through the mechanisms demonstrated in this figure. Nutrition may augment osteopathic techniques in improving overall health, and these ideas should be incorporated in osteopathic education systems.

In terms of cancer prevention, a nutritious plant‐rich diet plays a vital role in each of the five physiologic models of osteopathic medicine: the biomechanical, respiratory‐circulatory, neurological, metabolic‐energy, and behavioral models (Figure 4) [145]. For example, as mentioned previously, fruits and vegetables contain antioxidants and phytochemicals that bolster the immune system and reduce inflammation, relating to the respiratory‐circulatory model in osteopathic medicine which emphasizes the importance of lymphatic drainage and maximizing adequate oxygen and nutrient delivery [146]. The metabolic energy model emphasizes improving energy expenditure and decreasing allostatic load, and this model is important in cancer prevention because obesity is a known risk factor for multiple types of cancer and maintaining a healthy weight through proper nutrition has been shown to lower the risk of many cancers [147, 148]. The behavioral model emphasizes the importance of maintaining a healthy emotional, mental, and spiritual state of being and stresses assessing the mind, body, and spirit. The diagnosis of cancer can take a tremendous psychological toll on patients and family members, and depression or anxiety surrounding the diagnosis and treatment modalities may hinder survival and recovery [149]. This is particularly true of rare cancers that may occur in a younger demographic or are discovered at a later course of disease progression due to more limited screening methods [150]. Healthy eating patterns may reduce depression and suicide via altering neurotransmitter release and decreasing inflammation that may promote mental health problems, and good diet quality has been correlated with a significantly decreased risk of depression in cancer patients [151, 152, 153].

Osteopathic physicians often counsel patients on lifestyle modifications, including dietary changes, to promote overall health and reduce the risk of chronic diseases. They may also incorporate osteopathic manipulative treatment to support the body's self‐healing mechanisms. The American Association of Colleges of Osteopathic Medicine released a statement in April of 2024 addressing these ideas, and they plan to incorporate five guiding principles into osteopathic medical education: evidence‐based nutrition curriculum development, integration of nutrition across the medical education continuum, experiential learning opportunities and community engagement, interprofessional collaboration, and clinical training in nutritional counseling [154]. This approach will empower future physicians to use food as not only a source of energy, but also as a form of preventive medicine. By addressing nutrition as a fundamental aspect of preventive care, osteopathic medicine and its five physiologic models aligns with the broader understanding of how lifestyle factors can influence health outcomes, including cancer prevention.

In summary, the integration of nutrition into medical education, particularly within osteopathic medicine, plays a crucial role in the prevention and management of chronic diseases, such as cancer. While traditional medical education often falls short in providing adequate nutrition training, osteopathic medicine's holistic approach emphasizes the importance of a nutrient‐dense diet in maintaining overall health and preventing disease. By incorporating nutrition into the core curriculum, many osteopathic medical schools are preparing future physicians to effectively use nutrition as a tool for preventive care. This comprehensive approach is vital not only for preventing cancer but also for improving overall patient care and promoting long‐term health outcomes.

## 6 Conclusion, Challenges, and Future Directions

In conclusion, the detrimental impact of the SAD on cancer risk should be addressed in clinical medicine. The abundance of processed foods, saturated fats, refined sugars, low intake of fruits and vegetables, and epidemic of obesity contribute to an environment that favors cancer growth and progression. Various methods of cooking, such as frying, grilling, smoking, and cooking meats and other foods at high temperatures, generate carcinogens that may lead to DNA adducts, mutations, and lead to uncontrolled cell proliferation. Processed meats contain preservatives, such as nitrates and nitrites, that are converted to carcinogenic compounds when consumed in the diet. Conversely, embracing a plant‐rich diet that incorporates cancer‐protective foods, such as whole grains, fruits, vegetables, and legumes, may combat cancer‐promoting processes, making this dietary approach a cornerstone of cancer prevention. Research on cancer prevention and intervention with nutritional science has been limited because it requires many resources, has significant participant variation (sex, race, economic status, and fitness), recall bias, and other errors associated with collecting dietary data. Medical education on using nutrition as a means of cancer prevention is slim to none. Using diet as a means of preventing cancer particularly relates to osteopathic principles and practices as a holistic approach, and current medical education is lacking in this area.

Future research efforts should prioritize investigating dietary patterns in clinical studies to clarify the mechanisms by which nutrition influences cancer initiation, progression, and metastasis. Longitudinal studies assessing these dietary patterns and cancer risk should be performed as well as risk assessments to assess the advantages and disadvantages of limiting meat intake as a means of cancer prevention. These steps could allow a deeper understanding of how diet and cancer biology interact, and will allow refinement of current dietary recommendations to reduce cancer risk through nutritional modifications. The critical role of nutritional intervention in cancer therapy should be prioritized to avoid malnutrition and cachexia and alleviate cancer therapy‐induced symptoms. Additional research investigating specific diets and microbiome optimization that may augment concomitant therapies should be performed to guide evidence‐based recommendations. Last, integrating lifestyle medicine principles, such as nutrition, into clinical education and practice as well as public health initiatives may cause a shift toward a more preventive healthcare system that empowers patients to mitigate their risk of disease through sustainable lifestyle interventions. These ideas underscore the profound effect that everyday choices can exert on health outcomes and lie closely to the heart of osteopathic medicine by accentuating the interconnectedness of the body's structure and function to promote optimal health and wellness.

## Author Contributions

Taylor Collignon participated in conceptualization, performed data curation, prepared original draft, generated the figures and table, and revised the manuscript. Anupam Bishayee conceptualized this review, edited the manuscript, and supervised and administered the project. Both authors have endorsed the final manuscript for publication.

## Ethics Statement

The authors have nothing to report.

## Conflicts of Interest

The authors declare no conflicts of interest.

## Data Availability

The authors have nothing to report.
